# A novel NKG2A alpaca nanobody targeting immune checkpoint blockade for the treatment of malignant melanoma

**DOI:** 10.3389/fvets.2025.1571857

**Published:** 2025-04-30

**Authors:** Xiang Guo, Congfang Guo, Dongxiao Li, Yuting Bai, Mureed Abbas, Ruiwen Fan, Yiyan Zhao

**Affiliations:** ^1^Shanxi Bethune Hospital, Shanxi Academy of Medical Sciences, Tongji Shanxi Hospital, Third Hospital of Shanxi Medical University, Taiyuan, China; ^2^Tongji Hospital, Tongji Medical College, Huazhong University of Science and Technology, Wuhan, China; ^3^College of Animal Medicine, Shanxi Agricultural University, Taigu, China; ^4^Tianjin First Central Hospital, Tianjin, China; ^5^Department of Energy Chemistry and Materials Engineering, Shanxi Institute of Energy, Jinzhong, China; ^6^Research Institute of Applied Biology, Shanxi University, Taiyuan, Shanxi, China; ^7^College of Life Science, Shanxi University, Taiyuan, China

**Keywords:** alpaca nanoantibody, immune checkpoint inhibitors, immunotherapy, malignant melanoma, NKG2A

## Abstract

Alpacas belong to the Camelidae family. Antibodies produced through alpaca immunization are called nanoantibodies. Compared to traditional antibodies, nanoantibodies have several characteristics, including smaller molecular weight, stable structure, high homology with human antibodies, and suitability for prokaryotic expression. Malignant melanoma (MM) is a severe and aggressive form of cancer that affects both humans and animals. It commonly arises in the mucous membranes of the skin, nose, mouth, anus, and digestive tract, as well as in the choroid of the eyes. Multiple factors contribute to melanocyte carcinogenesis, including ultraviolet (UV) radiation, endocrine disorders, viral infections, immune deficiencies, and chemical carcinogens. At present, surgical resection remains to be the primary treatment for MM, although the prognosis is generally poor. However, targeted therapy and immune checkpoint inhibitors (ICIs) are increasingly employed in the clinical treatment of melanoma. NKG2A is an inhibitory receptor protein found on the surface of CD8^+^ T cells and natural killer (NK) cells. HLA-E ligands expressed on the surface of malignant melanoma cells can facilitate immune evasion by binding to the NKG2A receptor complex on immune cells. This interaction suppresses immune responses, enabling tumor cells to escape immune surveillance. Immunosuppressive antibody drugs work by blocking this recognition mechanism, thereby reactivating immune cells to target and destroy tumor cells. As such, NKG2A has emerged as a novel target for immunotherapeutic intervention. In this study, an alpaca-derived nanoantibody targeting NKG2A with high affinity was obtained from a melanoma-specific phage library display. Through induced expression and protein purification, a monoclonal nanobody strain expressing NKG2A was successfully isolated. This NKG2A-targeting nanobody demonstrates the potential for application in both the detection and treatment of MM.

## 1 Introduction

With advances in immunology and molecular biology, antibodies have become widely used in the diagnosis and treatment of diseases (1). In 1993, Hamers-Casterman et al. discovered that two kinds of immunoglobulins coexisted in the serum of the camel (*Camelus dromedarius*): One is the conventional 4-chain antibody consisting of two heavy chains and two light chains, and the other is a heavy-chain antibody (HCAb), which lacks light chains yet retains a broad antigen-binding repertoire (2). These unique HCAbs were later found in the serum of other animals, such as alpacas (3) and sharks (4).

Furthermore, the independently expressed variable domain of the heavy chain of the heavy-chain antibody (VHH) retains the ability to bind antigens, and its molecular size is only 1/10 that of traditional antibodies. It is also known as a single-domain antibody or nanobody (Nb). VHH consists of four framework regions (FR1, FR2, FR3, FR4) and three complementarity-determining regions (CDR1, CDR2, CDR3) that form the antigen-binding sites, with the leader area at the front and the hinge area at the back (2). Compared to traditional antibodies, single-domain antibodies have several distinctive characteristics, including small molecular weight, stable structure, high homology with human antibodies, and suitability for prokaryotic expression. These characteristics make them highly promising for applications in disease diagnosis, treatment, and commercialization (5, 6).

Malignant melanoma (MM) is a type of malignant tumor that originates from melanocytes located in the basal layer of the epithelium. It is characterized by its strong potential for invasion and metastasis, as well as a high mortality rate (7). In humans, melanoma commonly occurs in the skin, mucous membranes (including the digestive, respiratory, and genitourinary tracts), uvea, and pia mater, among other locations. In animals, melanoma is commonly found in the mouth, lips, and other areas where melanocytes are deposited (8). Several factors contribute to the carcinogenicity of melanocytes, including UV radiation, endocrine disorders, varying racial tolerance to external stimuli, viral infections, immune deficiencies, trauma sequelae, genetic factors, and chemical carcinogens (9–11). Accurate and standardized pathological diagnosis is crucial for the clinical treatment and prognosis of melanoma. In recent years, melanoma-targeted therapy and immunotherapy have demonstrated promising clinical efficacy. Additionally, advancements have been made in the field of pathological diagnosis of melanoma (12–14).

Melanoma is classified into various subtypes based on its site of occurrence. In cutaneous melanoma, mutations are commonly found in genes such as the *B-Raf proto-oncogene* (BRAF), *multiple tumor suppressor 1* (MTS1), *Neuroblastoma RAS viral oncogene homolog* (NRAS), and the *tumor protein p53* (*TP53*) gene. In acral melanoma, mutations occur in the *BRAF*, *NRAS*, and *neurofibromatosis type 1* (*NF1*) genes. In mucosal melanoma, mutations in the *SF3B1* gene impact those in the promoter of *telomerase reverse transcriptase* (*TERT*) (12). Similar to other cancers, melanoma can compromise the immune system of the affected body. Therefore, understanding how the body initiates and regulates the T-cell-dependent immune response is crucial for developing effective cancer treatments (13).

In recent years, significant advancements have been made in melanoma treatment, particularly in targeted therapy and immunotherapy, which have demonstrated promising clinical efficacy. Progress has also been observed in the pathological diagnosis of melanoma. In terms of pharmacological treatment, various chemotherapy drugs, such as dacarbazine, paclitaxel, and temozolomide, are commonly used. Although these drugs show some antitumor activity, both as monotherapies and in combination, their overall efficacy remains low and limited (14). The treatment of early-stage melanoma primarily involves surgery along with conventional drug treatments. However, treatment becomes significantly more challenging once the disease has metastasized (15).

NKG2 is a class of inhibitory or activating cell membrane receptors found on natural killer (NK) cells, comprising a total of seven members, all located in the NK gene complex on human chromosome 12p12.3-p13.1, and all featuring a C-type lectin-like structure (16). The NKG2A protein, a member of the NKG2 family, is a single transmembrane receptor protein with a relative molecular mass of 43KDa and is composed of 233 amino acids. NKG2A is also known as killer cell lectin-like receptor C1 (KLRC1). It is expressed in CD56dim NK cells, NK T cells (NKT), and CD8 + αβ T cell subpopulations. On the surface of human immune cells, the heterodimer complex NKG2A-CD94, formed by the NKG2A and CD94 molecules linked by disulfide bonds, recognizes the nonclassical major histocompatibility complex class I (MHC I) molecule HLA-E on target cells. HLA-E is normally expressed at low levels, but its expression increases on the surface of most tumor cells, thereby inducing a cascade of inhibitory signals that hinder NK cytotoxic activity and cytokine secretion (16–19). NKG2A is expressed in multiple tissues, including peripheral blood cells, bone marrow, and lymphocytes (20).

Immune checkpoint inhibitors (ICIs) are a popular target in the immunotherapy of malignant tumors. Since the Nobel Prize in Medicine in 2018 awarded recognition for a new method to treat cancer by inhibiting the negative regulation of tumor immunotherapy, the development of immunotherapy has significantly improved the overall survival rate of multiple myeloma (MM) patients. The emergence of ICIs has further promoted the advancement of MM immunotherapy; however, about half of patients still cannot benefit from it (21–23). Immune checkpoints are regulators of the immune system and are essential for maintaining autoimmune tolerance, as well as for regulating the duration and scope of immune responses in peripheral tissues.

Tumor cells can “hijack” these “checkpoints” and continue to activate through various strategies, suppressing the antitumor immune response and promoting tumor progression. Immune checkpoint inhibitors (ICIs) are a class of drugs that fight tumors or certain infectious diseases by blocking checkpoints and activating the immune system. In the context of MM therapy, ICIs primarily block the binding of inhibitory immune checkpoint receptors on the surface of T cells to ligands on tumor cells, thereby alleviating the suppression of immune activity against tumor cells and ultimately enhancing or activating the immune system to eliminate MM. Currently, the most extensively studied ICIs in MM clinical trials include programmed cell death protein-1 (PD-1) antibody and cytotoxic T lymphocyte-associated antigen-4 (CTLA-4) antibody (24–26). The main challenges faced by ICIs in the treatment of MM include insufficient response rates, complex resistance mechanisms, significant toxicity, and patient heterogeneity. Therefore, it is crucial to explore and develop novel and effective ICI antibodies for the treatment of MM. NKG2A is a promising inhibitory target in this approach.

In addition to NK cells, NKG2A is expressed on the surface of CD8^+^ T cells, Th2 cells, and NKT cells. Similar to the PD-1 receptor, NKG2A is a membrane protein receptor on immune cells that contains ITIMs. However, they recognize specific cells through distinct mechanisms. Tumor cells evade detection by the immune system through immune suppression mechanisms. Blocking this recognition pathway with a monoclonal antibody can activate immune cells to target and kill tumor cells. Studies suggest that the expression of the NKG2A protein receptor on the surface of CD8^+^ T cells is regulated by the tumor microenvironment (20, 27). CD8^+^ T cells in peripheral blood exhibit low expression of NKG2A, while most CD8^+^ T cells in the tumor display high expression of NKG2A (27).

However, NKG2A differs from PD-1 in that the PD-1 protein receptor primarily exists on the surface of certain T cells, whereas the NKG2A protein receptor is found on both some T cells and NK cells. NK cells and T cells employ different immune mechanisms, and NKG2A antibodies can activate both NK and T cells, which is the advantage of NKG2A as an immune target.

For the treatment of MM, cellular immunotherapy has ushered in a new era for tumor therapy. Although patients with MM exhibit a variety of responses to immunotherapy, including primary or acquired drug resistance, their survival time has been extended to some extent, and their quality of life has improved. In the future, immunotherapy will become the top priority in treating MM patients. The key to immunotherapy lies in understanding how tumor cells evade immune system surveillance, identifying specific targeted immune markers, and adopting the most appropriate treatment strategies to enhance patient outcomes, including overall survival (OS) and quality of life.

In this study, we developed and evaluated a novel alpaca NKG2A nanobody for the treatment of malignant melanoma in mice, providing a new approach and perspective on the treatment of malignant melanoma.

## 2 Materials and methods

### 2.1 Validation of B16-F10 nanobody library

The melanoma cell nanobody library was used for NKG2A nanobody screening. The capacity and abundance of the antibody library are critical for the subsequent screening of target antibodies, and they are positively correlated with the ability to identify specific antibodies to a certain extent (28). VHH fragments are easily lost during the construction of phage display libraries, which affects the library capacity and makes subsequent specific antibody screening difficult (29). Therefore, identifying existing antibody libraries before screening is necessary. When the antibody library’s capacity is approximately 1×10^7^, it meets the requirements for subsequent screening (29). In this study, the B16-F10 melanoma nanobody library, provided by the Alpaca Nanobody Laboratory at the College of Veterinary Medicine of Shanxi Agricultural University, was employed to screen for anti-NKG2A antibodies (28).

Assay of library capacity: The original bacterial solution used to determine library capacity was diluted in a gradient, with dilution ratios ranging from 10^−1^ to 10^−6^. Each plate was coated with 100 μL, and each concentration was cultured on 2 × YTAG solid culture plates overnight at 30°C. The next day, the number of colonies on each gradient storage plate was counted, and the storage capacity of the nanobody library was calculated.

Identification of library abundance: The bacterial solution used to determine the abundance of the library was diluted in a gradient, with a dilution concentration ratio of 10^−4^–10^−8^. Each plate was coated with 100 μL of the diluted solution, and each concentration was cultured on 2 × YTAG solid culture plates overnight at 30°C. The following day, the number of colonies on each gradient storage plate was counted, and the abundance of the nanobody library was calculated.

### 2.2 Preparation and titration of helper phage

Helper phage M13KO7 (Bio-View Shine Biotek) was streaked on 2 × YT solid medium. Mix 0.5 mL of log-growing TG1 solution (D600 = 0.6) with 4.5 mL of 2 × YT semi-solid agar (0.7% agar) and pour the upper agar diagonally into the petri dish in reverse order of streaking. After incubating overnight at 37°C, the plaque was inoculated in 100 mL of 2 × YTAK (100 μg/mL ampicillin and 50 μg/mL kanamycin) overnight. On the third day, the supernatant was filtered through a 0.45 μm filter membrane after centrifugation at 16000 rpm at 4°C and stored at 4°C for future use. The prepared bacteriophage was gradient diluted to 10^−6^ to 10^−12^, and the TG1 bacteria solution in the logarithmic growth phase was added to each tube along with 100 μL of gradient diluted bacteriophage, then cultured on a constant temperature shaking table at 200 rpm at 37°C for 30 min. Moreover, 3 mL of upper agar was added to each tube and poured onto a 2 × YT solid culture plate, followed by overnight incubation at 37°C. The phage titer was calculated based on the number of plaques observed the next day.

### 2.3 Screening of antigen-positive recombinant phages in enriched clones

NKG2A polypeptide (Bioss bs-2411p) was diluted to a final concentration of 20 μg/mL, wrapped in an immune tube, and stored at 4°C overnight for further use. The 500 μL phage library was dissolved in 100 mL of 2YTAG solution medium and incubated at 37°C with shaking at 200 r/min until the value of OD600 nm ranged from 0.4 to 0.6. After adding 100 μL of helper phage and shaking at 37°C at 200 r/min for 30 min, the supernatant was discarded, and 100 mL of 2YTAK liquid medium was added to re-suspend the precipitation, followed by overnight shaking at 30°C at 200 r/min.

The following day, TG1 was added to 30 mL of 2YT and cultured at 37°C at 200 r/min until the optical density (OD) reached 0.4. After centrifugation at 4°C at 11000 r/min for 10 min, the supernatant was discarded, and 1/5 volume of PEG/NaCl was added and placed on ice for 70 min. The pellet was resuspended in 2.4 mL of sterile PBS after centrifugation. The supernatant was recovered and added to an immunocoated tube that had been previously coated with 20 μL/mL of NKG2A polypeptide. After sealing at 37°C for 1 h, rinsing with PBS, and adding triethanolamine, the tube was shaken gently for 15 min, followed by the addition of Tris–HCl to neutralize the solution. The mixture was then coated on a 2YTAG plate and cultured at 30°C overnight to obtain a primary library. After five rounds of screening, 192 monoclonal colonies were picked from the library plates. The enzyme-linked immunosorbent assay (ELISA) was performed as described previously (30). Briefly, the phage supernatant treated with blocking solution was added to ELISA plates (Corning, SNY, New York, NY, USA) coated with NKG2A recombinant protein for specific binding, followed by incubation with HRP-conjugated anti-M13 phage antibody (1:10000, Sinobiological, Beijing, China) as the secondary antibody for affinity identification and screening. The ELISA results of the clones were analyzed, with a ratio of positive to negative values (P/N) ≥ 2.1 set as the threshold for positive affinity. The positive strains were inoculated into 5 mL of 2 × YTAG medium, incubated overnight at 37°C, and then sequenced.

### 2.4 Recombinant expression, purification, and characterization of the NKG2A nanobody

Codon optimization of nanobody sequences was conducted, followed by the design of primers. The 5′ and 3′ ends were incorporated into the BamHI and SalI enzyme cleavage sites, respectively, and then ligated into the Pcold prokaryotic expression vector. As previously described, the recombinant plasmid was sequenced and transformed into *Escherichia coli* BL21 (DE3) competent cells (TransGen), which were cultured at 37°C and induced with 0.4 mM isopropyl *β*-D-1-thiogalactopyranoside (IPTG). The bacteria were harvested by centrifugation and disrupted using ultrasound. After centrifugation, the supernatant was collected, and the samples were analyzed by SDS-PAGE electrophoresis (15% (w/v) polyacrylamide gel).

The supernatant was filtered using a 0.22 μm filter, purified with an Ni-NTA affinity column (GE Healthcare), eluted via gradient elution, and the elution samples were collected and confirmed by Western blotting. Following transfer onto a polyvinylidene difluoride (PVDF) membrane, the protein was incubated with an HRP monoclonal antibody (1:25000) targeting the His tag at 37°C and then visualized with CDP-Star chemiluminescent substrate (Tropix). The primers used in this study are listed in Table 1.

**Table 1:** Primers for VHH PCR.

Primer names	Sequence of primers (5′-3′)
VHH-F	GTGA**GGATCC**CAGGTGCAGCTGGTGGAGACTGGGGGAGGCT TGGTACAGCC
VHH-R	TCTG**AGTCGACT**TATGACCAGACGGTCACCTGGGTCCCCT

The bold letters represent the digestion sites.

### 2.5 Detection of the NKG2A nanobody binding to the antigen

The binding of the NKG2A nanobody to the antigen was detected using indirect ELISA. An ELISA plate was coated with NKG2A polypeptide (2 μg/well), followed by incubation with NKG2A VHH (containing His-tag, 10 μg/well) as the primary antibody and mouse anti-HIS-HRP monoclonal antibody (1:10000) as the secondary antibody. The ELISA stop solution was added after the chromogenic reaction, and the A450 absorption value was measured using a microplate reader. Anti-NKG2A antibody (Bioss, bs-2411R) served as a positive control, while PBS was used as a negative control instead of the NKG2A polypeptide antigen. BSA was included as a blank control. Three replicates were performed in each group.

Western blot detection of nanobody: The mouse-immortalized macrophage RAW264.7 was lysed and then subjected to SDS-PAGE electrophoresis. Purified NKG2A nanobody served as the primary antibody, while HRP His Tag acted as the secondary antibody for Western blot analysis to detect the expression of NKG2A protein in macrophage RAW264.7. 293T cells were used as the negative control.

Immunohistochemical detection of nanobodies: As previously described (30), paraffin sections of mouse spleen were dewaxed, antigen-repaired, and incubated in 3% H_2_O_2_ at room temperature for 30 min. After washing with PBS, they were blocked in 5% BSA at 37°C for 1 h, followed by overnight incubation with purified nanobodies at 4°C and subsequent incubation with His-tag antibodies at 37°C for 1 h. After washing with PBS, color development was performed using the DAB Kit, and the sections were counterstained with hematoxylin. The sections were dehydrated and sealed in neutral resin, and the expression distribution of NKG2A in mouse spleen tissues was observed via microscopy (Olympus, Shinjuku-ku, Japan).

### 2.6 Effect of NKG2A nanoantibody on melanoma proliferation in mice

A total of 20 male, 6-week-old Balb/c mice were divided into four groups to establish the mouse model. The Balb/c mice were injected subcutaneously with 1 × 10^^6^ B16-F10 cells. After 7 days, when the mouse tumor model had formed, the drug administration experiment was conducted. Mice in groups 1, 2, and 3 were injected with purified nanobody at doses of 30 μg, 50 μg, and 70 μg, respectively, while the control group received an equal volume of normal saline. Tumor sizes of the mice were measured every 3 days for 30 days using calipers. The tumor volume was calculated using the following formula: tumor volume = (length) × (width)^2^ × 0.5. When a tumor reached ethical limits, all mice were euthanized, and the tumors were collected and weighed.

### 2.7 Statistical analyses

Data were presented as mean ± SEM, with error bars indicating SEM. *p* values were calculated using either unpaired or paired two-tailed Student’s *t-test,* with *p < 0.05, **p < 0.01, and ***p < 0.001. All analyses were conducted using GraphPad Prism software, and all experiments were performed three times.

## 3 Results

### 3.1 Validation of the anti-melanoma nanobody library

On the phage library capacity assay plate (10^−6^ dilution), 82 monoclonal colonies were found (Supplementary Figure S1), resulting in a capacity of 82 ÷ (100 × 10^−6^) × 20 × 10^3^ = 1.64 × 10^10^ colonies/mL. On the phage library abundance assay plate (10^−8^ dilution), 108 colonies were identified (Supplementary Figure S2), and the abundance of the nanobody phage library was calculated as 108 ÷ (50 × 10^−8^) × 10^3^ = 2.16 × 10^11^ colonies/mL. The results indicated that the B16-F10 nanobody library exhibited good capacity and abundance, with an insertion rate of the VHH fragment at 96% (29), making it suitable for screening experiments of NKG2A-specific single-domain antibodies.

### 3.2 Screening of anti-NKG2A nanobody strains

Positive strains were screened using ELISA, and the reactivity of 192 monoclonal phage supernatants with the NKG2A polypeptide was assessed via indirect ELISA. Based on the indirect ELISA results, nine monoclonal strains were selected, all of which exhibited good reactivity with the NKG2A polypeptide (Table 2).

**Table 2:** Results of single-domain antibody to NKG2A screened by ELISA assay.

Positive clone number	Experimental group	Control (BSA)	Negative control (PBS)
NKG2A-VHH-A3-1	0.365	0.272	0.073
NKG2A-VHH-D2-1	0.355	0.279	0.069
NKG2A-VHH-D3-1	0.366	0.271	0.067
NKG2A-VHH-H4-1	1.747	1.382	0.067
NKG2A-VHH-G7-2	0.311	0.152	0.082
NKG2A-VHH-G8-2	0.769	0.224	0.077
NKG2A-VHH-H2-2	1.414	0.267	0.064
NKG2A-VHH-H7-2	0.354	0.233	0.082
NKG2A-VHH-H12-2	0.394	0.166	0.079

**Figure 1:**
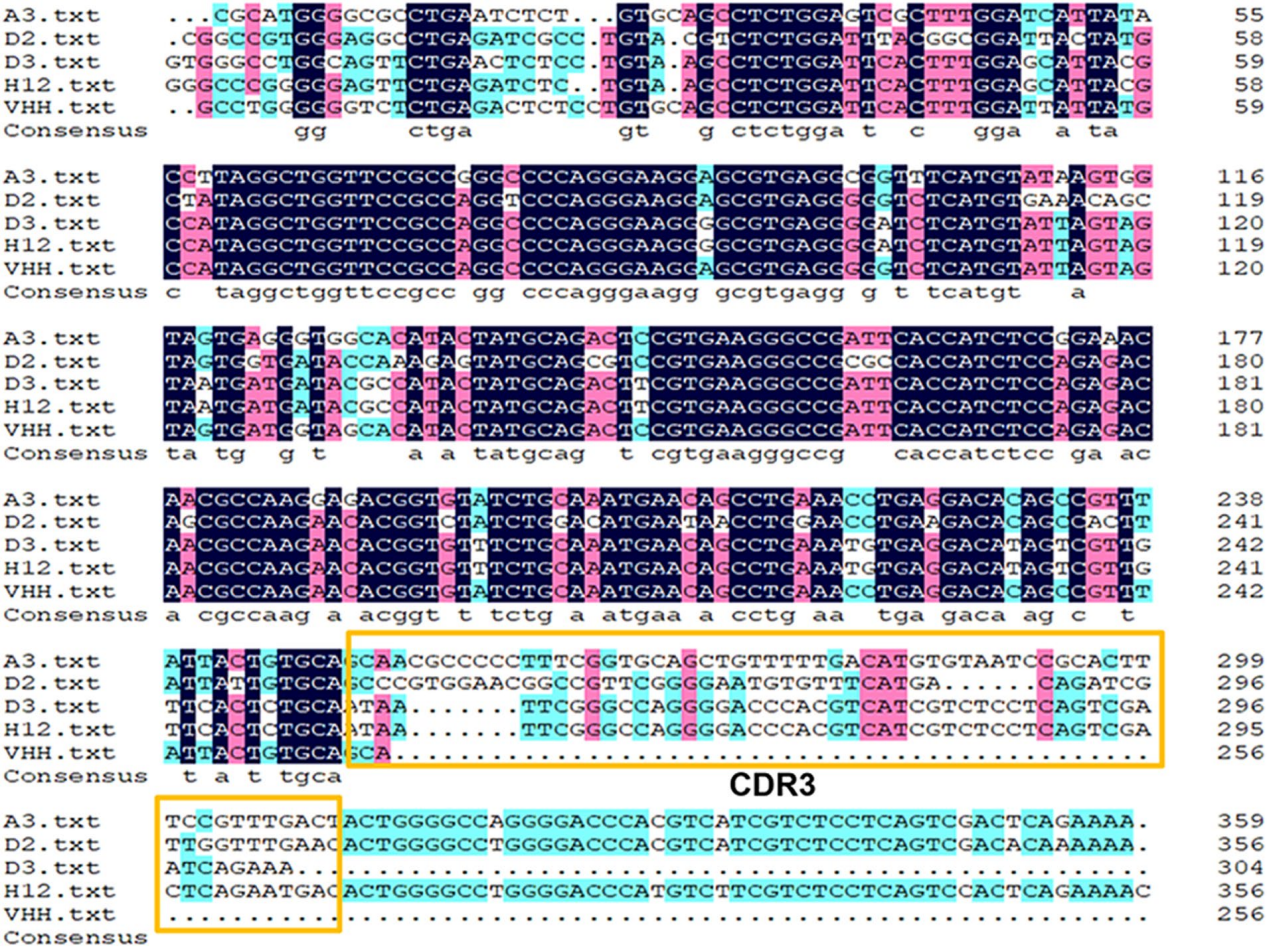
Alignment of the NKG2A VHH sequence with the reference sequence from GenBank. The complementarity-determining region 3 (CDR3) motif is highlighted with yellow boxes.

The nine strains mentioned above were sequenced, and four strains—NKG2A-VHH-A3-1, NKG2A-VHH-D2-1, NKG2A-VHH-D3-1, and NKG2A-VHH-H2-2—were screened. The nucleotide sequencing results were then compared with the VHH reference sequences in the NCBI Gene Bank; the results indicated that the similarity between NKG2A-VHH and VHH sequences reached 91.02% (Figure 1).

Protein expression screening: The Pcold vector with a His label is a modified *E. coli* protein expression vector developed in our laboratory. The VHH sequence was transferred to the Pcold vector via double-enzyme subcloning using BamHI and SalI. Western blotting was conducted using a His-labeled antibody as the primary antibody, with only two strains (NKG2A-VHH-A3-1 and NKG2A-VHH-D2-1) showing positive bands below 15KDa (Supplementary Figure S1). Sequence alignment of the amino acid sequences of the VHH fragment revealed that the amino acid sequence of NKG2A-VHH-A3-1 consists of 131 amino acids (Table 3), which includes four complete framework regions (FR1-4) and three complementarity determination regions (CDR1-3). CDR3 serves as the antigen-specific recognition region, containing 22 amino acids. The amino acid sequence of NKG2A-VHH-D2-1 consists of 112 amino acids (Supplementary Table S1), which includes three framework regions (FR1-3) and three complementarity determination regions (CDR1-3). CDR3 is an antigen-specific region comprising 18 amino acids.

**Table 3:** Frames of amino acid sequence of NKG2A VHH.

Region	Sequence
Frame-1	QLVESGGSLVQPGGSLRLSCAAS
CDR-1	GVALDHYT
Frame-2	LGWFRRAPGKEREAVS
CDR-2	CISGSEGGT
Frame-3	YYADSVKGRFTISGNNAKETVYLQMNSLKPEDTAVYYC
CDR-3	AATPPFGAAVFDMCNPHFPFDY
Frame-4	WGQGIQVTVSSAHH

Amino acid sequence analysis: An important factor affecting the expression level of recombinant DNA is the frequency of rare codons. The frequency of rare codon usage in *E. coli* expressing the NKG2A VHH single-domain antibody was predicted. The results indicated that most codons in NKG2A-VHH-A3-1 and NKG2A-VHH-D2-1 (Supplementary Figure S3) were highly expressed, and the occurrence of rare codons was low, suggesting that proteins could theoretically be expressed without issues.

Before protein expression, it is also necessary to analyze the amino acid sequence to identify any potential transmembrane sequences. This step helps prevent unintended protein expression on the cell membrane. TMHMM 2.0^1^ was used to analyze whether there were transmembrane sequences in the amino acid sequences of VHH from these two strains. As shown in Supplementary Figure S4, the VHH of both strains had no transmembrane structure. Before protein expression, strains with good hydrophilicity were screened according to the amino acid sequence. As shown in Supplementary Figure S5, the amino acid sequence of VHH expressed by these two strains demonstrated good hydrophilicity, ensuring the stability of their aqueous phase structure and providing conditions for protein expression.

**Figure 2:**
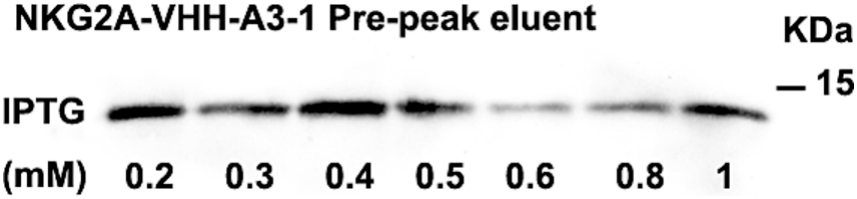
Expression of VHH in NKG2A-VHH-A3-1 at varying concentrations of IPTG, as analyzed by western blotting.

**Figure 3:**
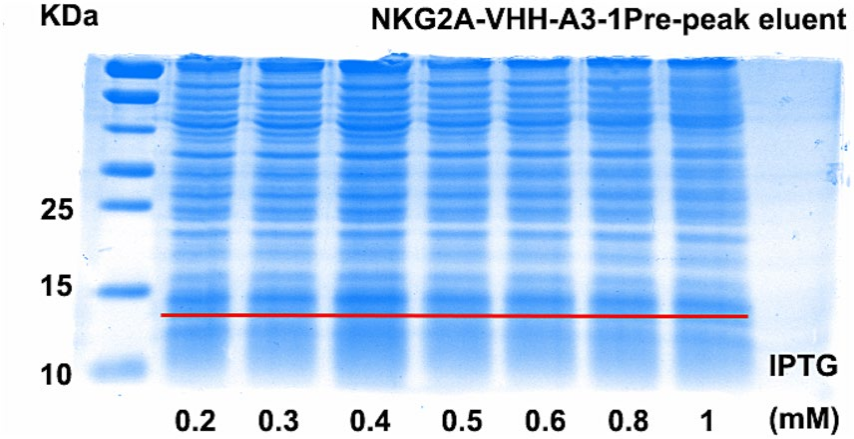
Expression levels of VHH in NKG2A-VHH-A3-1 at various IPTG concentrations, assessed by Coomassie Blue staining.

The optimal conditions for inducing NKG2A VHH expression were screened using Western blotting and Coomassie blue staining. Two temperatures, 15°C and 20°C, were selected for screening, while other induction parameters were kept constant at 0.4 mM IPTG, 120 rpm, and 20 h. As shown in Supplementary Figure S1, the protein expression of the NKG2A-VHH-A3-1 strain was higher than that of the NKG2A-VHH-D2-1 strain, particularly at 20°C. At 15°C, the expression of VHH in both the supernatant and precipitate of both strains increased, whereas the expression of NKG2A-VHH-D2-1 decreased (Supplementary Figure S6). Thus, the optimal induction temperature was determined to be 15°C. Subsequently, IPTG was tested at seven concentration gradients: 0.2 mM, 0.3 mM, 0.4 mM, 0.5 mM, 0.6 mM, 0.8 mM, and 1 mM, with other induction conditions unchanged (15°C, 120 rpm, 20 h). The results of Western blotting indicated that the optimal IPTG induction concentration for NKG2A-VHH-A3-1 was 0.4 mM, while for NKG2A-VHH-D2-1, the optimal concentrations were 0.3 mM and 0.5 mM (Figure 2 and Supplementary Figure S7). Coomassie blue staining results showed that the optimal IPTG-induced concentration for NKG2A-VHH-A3-1 was 0.4 mM, and for NKG2A-VHH-D2-1, it was 0.5 mM (Figure 3 and Supplementary Figure S8), although the expression level of NKG2A-VHH-D2-1 was extremely low. Therefore, the NKG2A-VHH-A3-1 strain (referred to as NKG2A VHH in subsequent studies) was preferred for further experiments.

**Figure 4:**
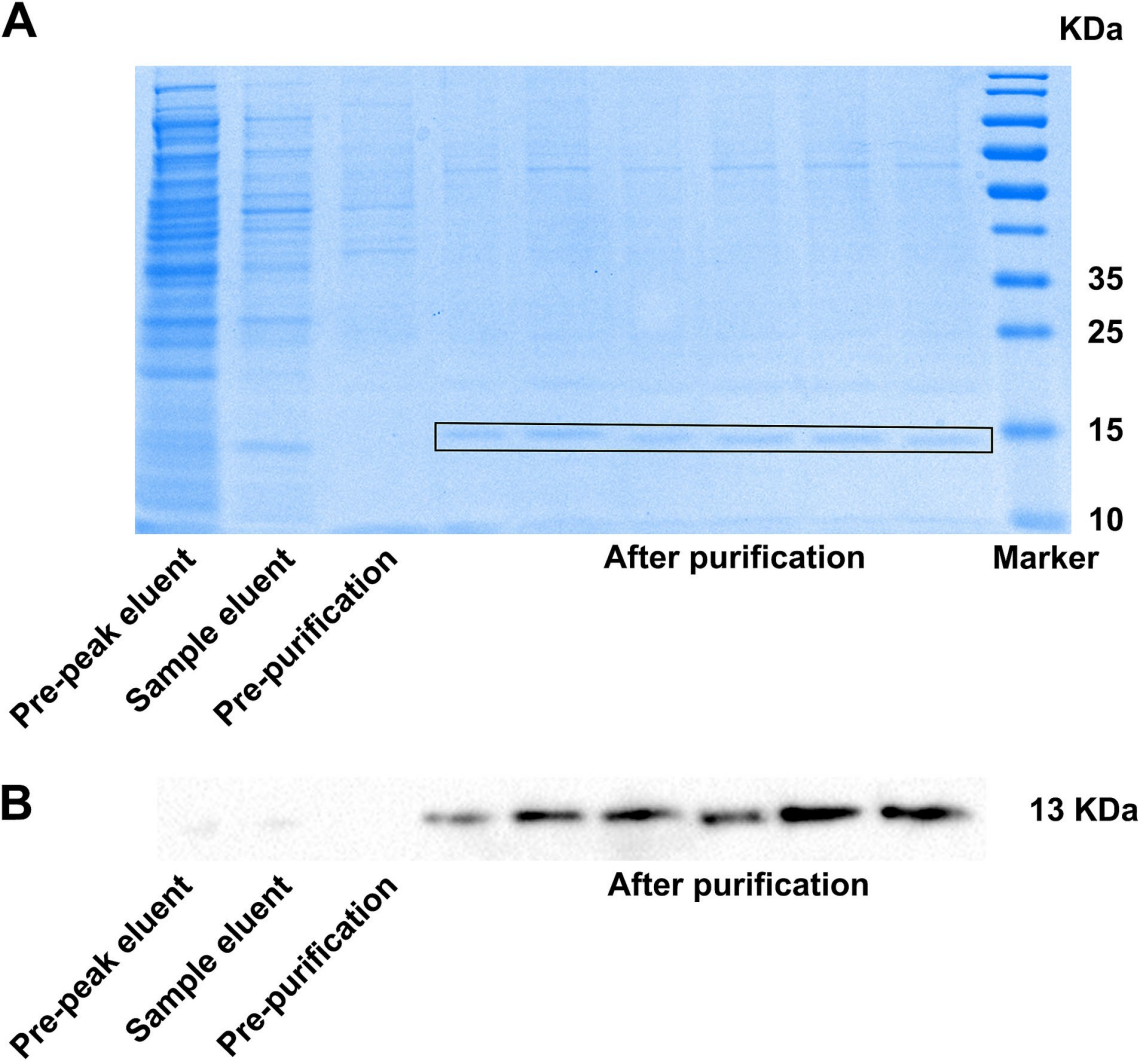
**(A)** SDS-PAGE (heated CBB staining) results and **(B)** Western blotting results showed the results of NKG2A VHH affinity chromatography.

After affinity purification, the bacterial solution supernatant, post-column solution, binding solution, and 13% imidazole eluent were collected. The purification effect of the affinity purification was verified by Western blotting and Coomassie blue staining. The Western blotting results showed that the positive bands in the 13% imidazole eluant were significantly more prominent compared to those in the supernatant and post-column solution after cell lysis (Figure 4A), indicating that the VHH-A3-1 nanobody had a better elution effect in 13% imidazole and exhibited a concentrated effect. The results of Coomassie blue staining showed that the 13% imidazole eluant had clear target bands below 15 KDa, and the bands were relatively uniform (Figure 4B), suggesting that the NKG2A VHH nanobody demonstrated good affinity chromatography under 13% imidazole conditions.

### 3.3 Detection of NKG2A VHH binding

**Figure 5:**
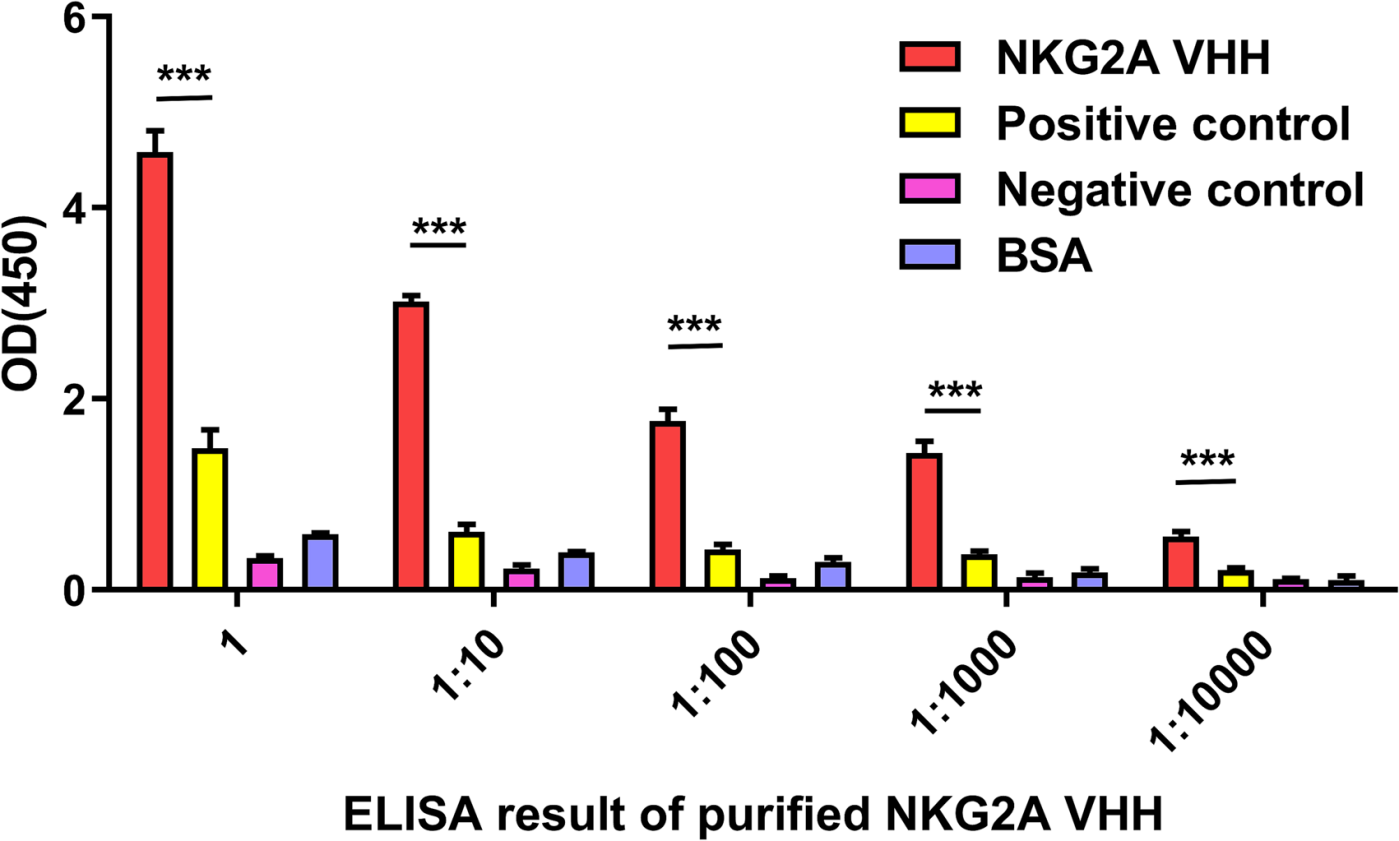
The affinity between NKG2A VHH and the antigen was detected using indirect ELISA. An anti-NKG2A antibody (Bioss, bs-2411R) served as a positive control, while PBS acted as a negative control instead of the NKG2A polypeptide antigen. BSA was used as a blank control. Three replicates were established for each group. ***p < 0.001.

The affinity between NKG2A VHH and the antigen was detected using an indirect ELISA. After the chromogenic reaction, the value of A450 in the NKG2A-coated well was significantly higher than that of the control group, indicating a strong binding activity of the antibody to the antigen (Figure 5).

**Figure 6:**
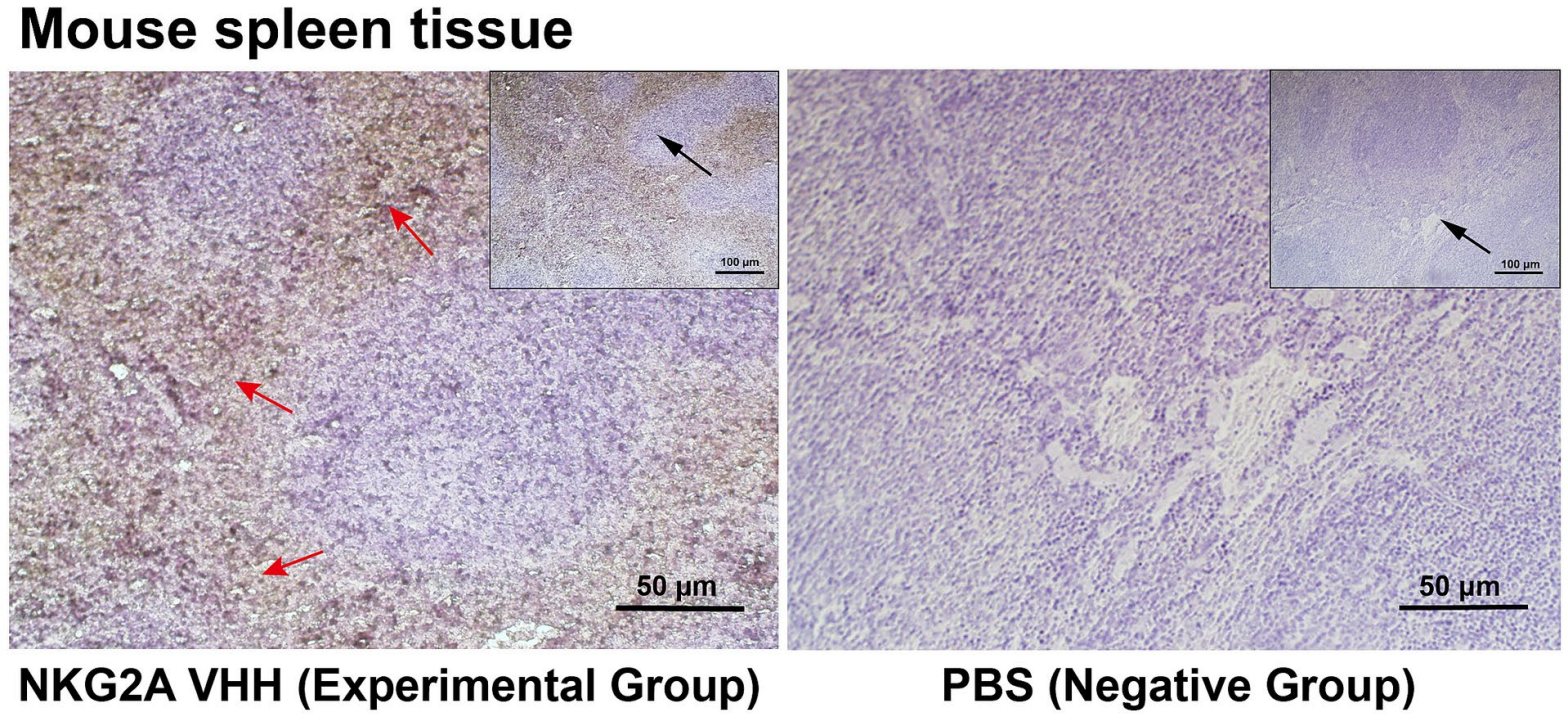
NKG2A VHH was used to assess the distribution of NKG2A in tissues. PBS served as a negative control. The NKG2A VHH is located in NK cells in the red pulp region of spleen tissue. Red arrows indicate the red pulp region, while the black arrow represents the zoom.

NKG2A is expressed in CD56hi natural killer cells (NK), natural killer T cells (NKT), and CD8^+^ αβ T cells; therefore, the spleen was selected as the tested tissue. Purified NKG2A VHH nanobody was used as the primary antibody in immunohistochemical experiments. As shown in Figure 6, the spleen tissue in the experimental group exhibited stronger immune signals compared to the control group, in which PBS was used instead of the primary antibody. These results suggest that the NKG2A VHH nanobody can be applied for tissue localization and relative quantitative detection of the NKG2A protein.

### 3.4 Preliminary study on inhibition of melanoma growth by NKG2A VHH in mice

**Figure 7:**
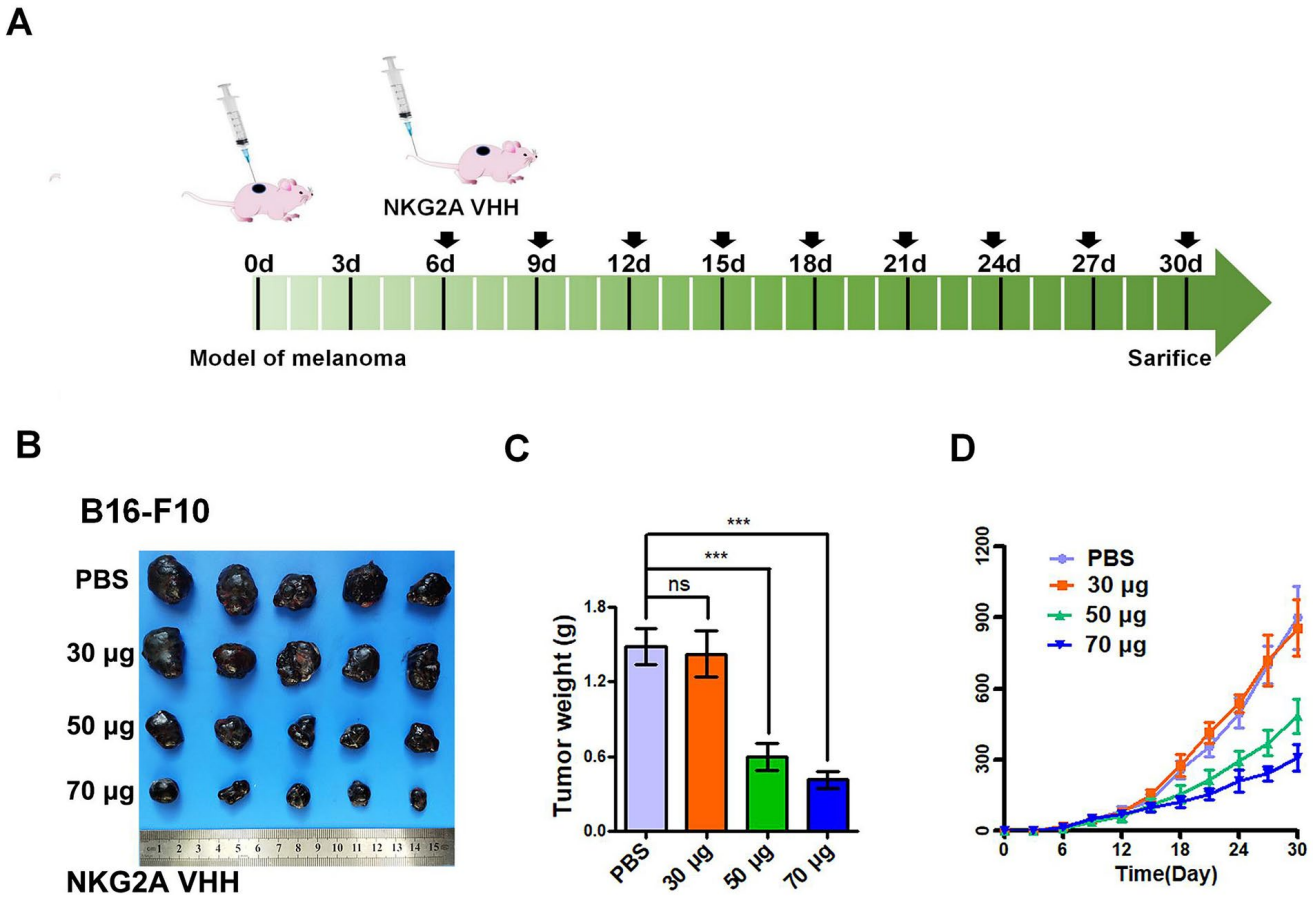
The effect of NKG2A VHH nanobody at different doses on melanoma growth in the Balb/c mouse model. **(A)** Schematic diagram of NKG2A VHH for the treatment of malignant melanoma in mice. **(B)** Compared to the PBS control group, the 30 μg dose had a minimal effect, the 50 μg dose showed a noticeable effect, and the 70 μg dose demonstrated a significant effect. **(C)** Tumor weights in the control group were compared to the experimental group after drug treatment. ***p < 0.001. **(D)** Growth rates of allogeneic tumors in Balb/c mice at various doses during tumor volume assays from 0 to 30 days.

The NKG2A VHH nanobody exhibited an inhibitory effect on the growth of mouse tumors. When 30 μg of NKG2A VHH was administered to mice, no significant change was observed in the growth rate or final size of the allogeneic tumors compared to the control group. However, at a dose of 50 μg, tumor growth in mice was significantly inhibited, with the most pronounced effect observed at a dose of 70 μg (Figures 7A–D). It was determined that NKG2A VHH possesses biological activity in mice, suggesting that an appropriate amount of NKG2A VHH could block the NKG2A receptor, inhibit the binding of the Qa-1b ligand to the NKG2A receptor directly, and thus activate the immune system to target and kill tumor cells.

## 4 Discussion

In this study, we constructed a phage display library of a nanobody against human melanoma generated in alpacas. As a result, we obtained a high-affinity nanobody against NKG2A, specifically NKG2A VHH, indicating that the blockade of NKG2A signaling is an effective therapeutic approach for the clinical treatment of melanoma. Furthermore, the anti-NKG2A nanobody, with its strong affinity, represents a potential new antagonist for melanoma therapy and could serve as an imaging agent for related research.

Immune checkpoint inhibitors have revolutionized cancer treatment (29). NKG2A is an inhibitory receptor protein containing an immunoreceptor tyrosine-based inhibition motif (ITIM) and is expressed in both T cells and NK cells (18, 31). The inhibitory signal is transduced via the protein tyrosine phosphatase SHP-1 (32). While the baseline expression of NKG2A in CD8^+^ T cells in human peripheral blood is low, it can be upregulated upon cellular activation (32). Studies have shown that NKG2A is expressed on both NK cells and T cells in various tumors, with its ligand HLA-E frequently overexpressed in tumor cells (29, 33). This conserved expression pattern may result from the reliance of HLA-E expression on various peptides, including precursor peptides derived from HLA-A, -B, or -C molecules (34, 35).

The application of immune checkpoint inhibitors has significantly improved clinical manifestations and extended progression-free survival in cancer patients (29). However, further research is necessary to manage the intensity of their toxicity and broaden patient coverage (18, 36, 37). Achieving this goal requires exploring new immune targets alongside the existing star immune targets, PD-1 and CTLA-4, as well as combining drugs to promote an effective immune response to cancer (38). To date, most immune-modulatory strategies have focused on enhancing the T cell immune response *in vivo* (39, 40), but there has been a growing interest in utilizing relatively underexplored NK cells for therapeutic interventions in recent years (41). Employing NK cells *in vivo* to exert immune function aims to initiate a multi-layered immune response, ultimately assembling various immune cells, including T cells, to generate a multifaceted and durable immune response to tumors (32, 42).

The binding of the NKG2A receptor to the ligand Qa-1b is inhibited by a monoclonal antibody drug that can enhance the immune activity of human natural killer (NK) cells and CD8^+^ T cells (18). In mouse tumor models, Monalizumab (an antibody to NKG2A), which is a humanized antibody vaccine against the NKG2A cell membrane receptor protein (18, 43), functions to enhance the immune activity of NK cells and CD8^+^ T cells against various tumor cells and also increases the survival time of patients (27, 44, 45). Monalizumab plays a role in preventing the NKG2A/CD94 receptor protein complex on the surface of immune cells from binding to the HLA-E ligand-protein complex on the surface of target cells (45–47). Preliminary clinical studies have been conducted in rheumatoid arthritis, metastatic colorectal cancer, head and neck cancer, and gynecological malignancies (18, 29, 48–50), yielding promising results in clinical trials. However, the clinical efficacy of NKG2A immune checkpoint inhibitors is still inconclusive (29, 43). In conclusion, existing animal experiments and clinical trials have shown that NKG2A inhibitors could activate the immune response of NK cells and T cells against tumor cells (27, 48–50).

**Figure 8:**
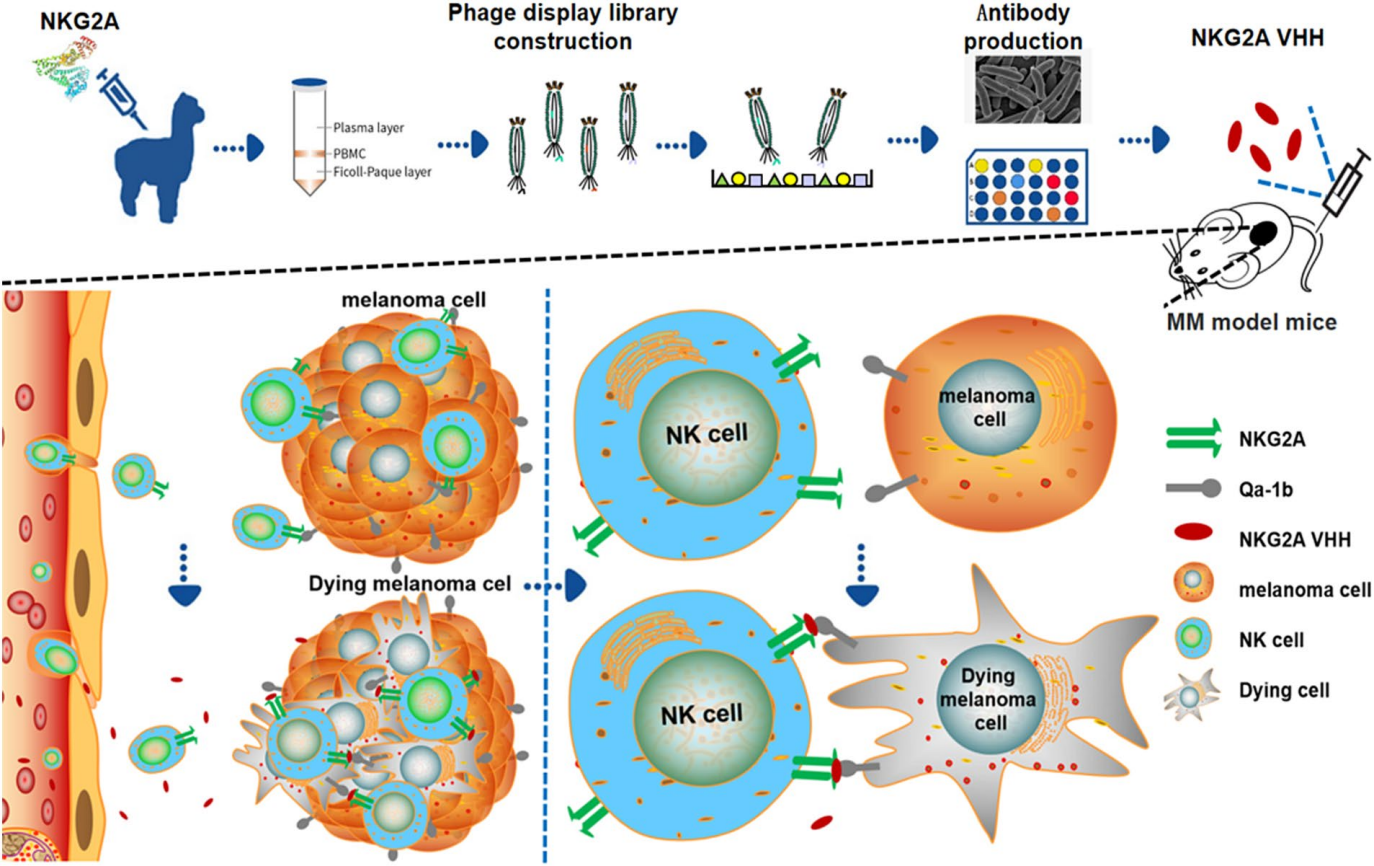
Model for the preparation of novel nanoantibody drugs for NKG2A VHH and treatment for MM.

Based on the molecular mechanisms involved in melanoma control, several monoclonal antibodies have been successfully applied in clinical practice. However, their therapeutic application remains limited by challenges such as solid tumor penetration and high manufacturing costs. In addition to the well-known PD-1 and CTLA-4 target mechanisms, the action mechanism of the NKG2A antibody represents a new checkpoint inhibitory pathway, promoting the immune response of antitumor cells by enhancing the activity of T cells and NK cells. This could complement the immunotherapy of primary PD-1 and CTLA-4 targets (51). New immunotherapy drugs targeting NKG2A are expected to be developed as innovative antibody treatments to inhibit the progression of malignant melanoma in both humans and animals. Compared to PD-1/PD-L1 targets, NKG2A immune checkpoint inhibitors are anticipated to bring new hope to patients’ treatment. This study highlights the potential of the NKG2A nanoantibody in overcoming the immunosuppressive effects of melanoma by activating natural killer (NK) and CD8^+^ T cells in both mice and humans, thereby enhancing the immune response against tumor cells. The findings suggest that NKG2A VHH holds significant promise as a novel antibody-based drug for the treatment of melanoma (Figure 8).

## Data Availability

The original contributions presented in the study are included in the article/Supplementary material, further inquiries can be directed to the corresponding authors.

## Footnotes

^1^ https://services.healthtech.dtu.dk/service.php?
