# Supplementary material for: A novel NKG2A alpaca nanobody targeting immune checkpoint blockade for the treatment of malignant melanoma
Source: Front Vet Sci. 2025 Apr 30;12:1571857. doi: 10.3389/fvets.2025.1571857 (PMC12076520; doi:10.3389/fvets.2025.1571857)
Supplement: Supplementary file 1 [file Data_Sheet_1.docx]

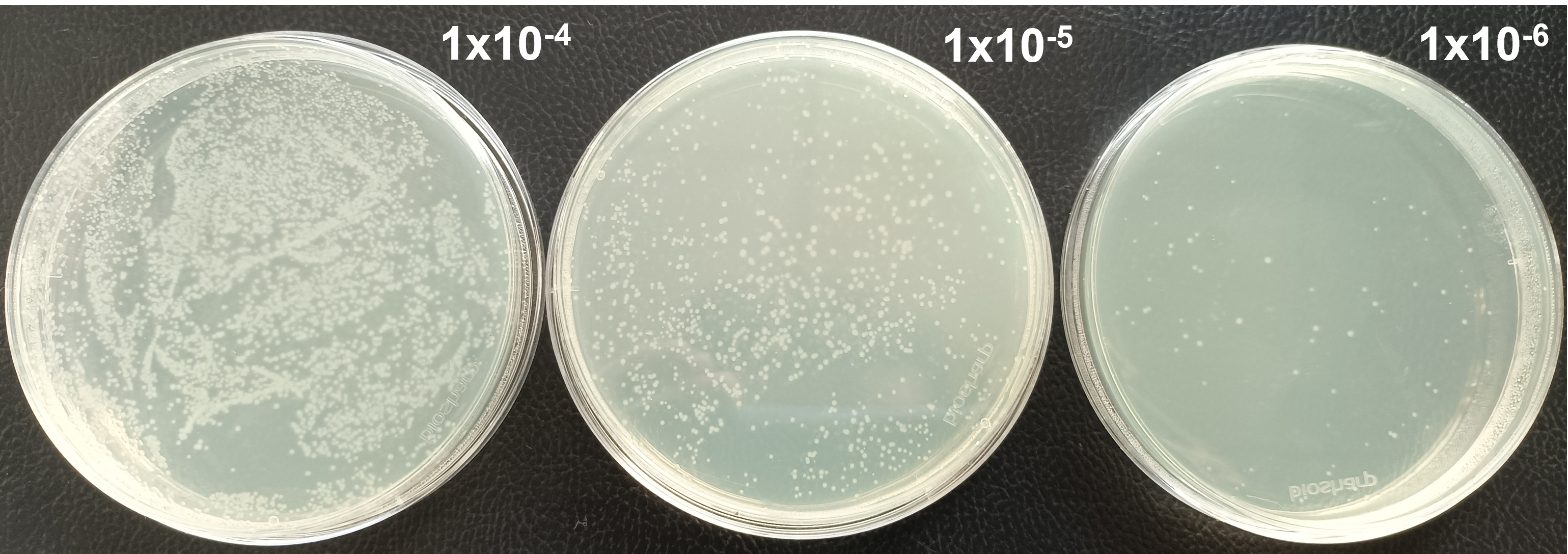


Fig. S1 Capacity of anti-Melanoma nanobody phage immune library


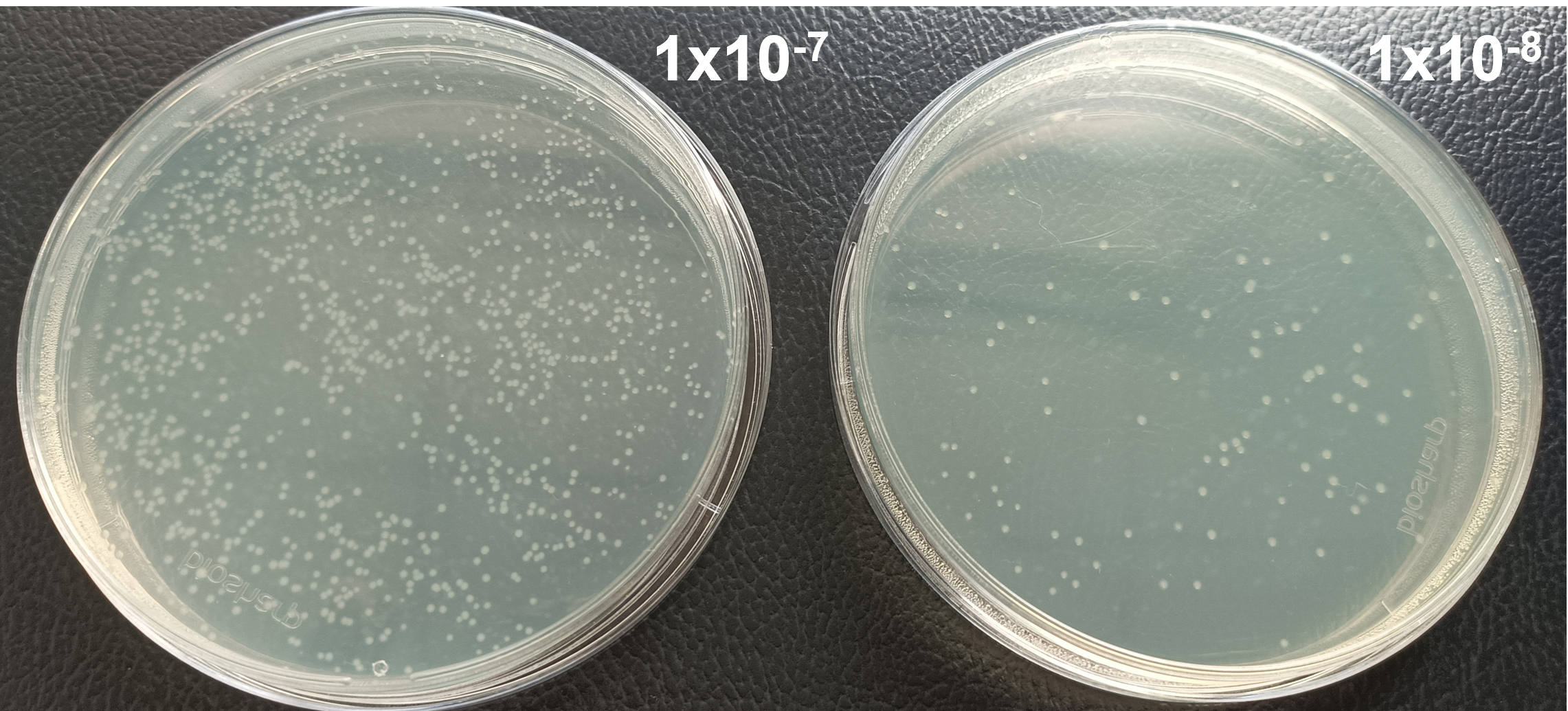


Fig. S2 Abundance of Anti-Melanoma nanobody phage immune library


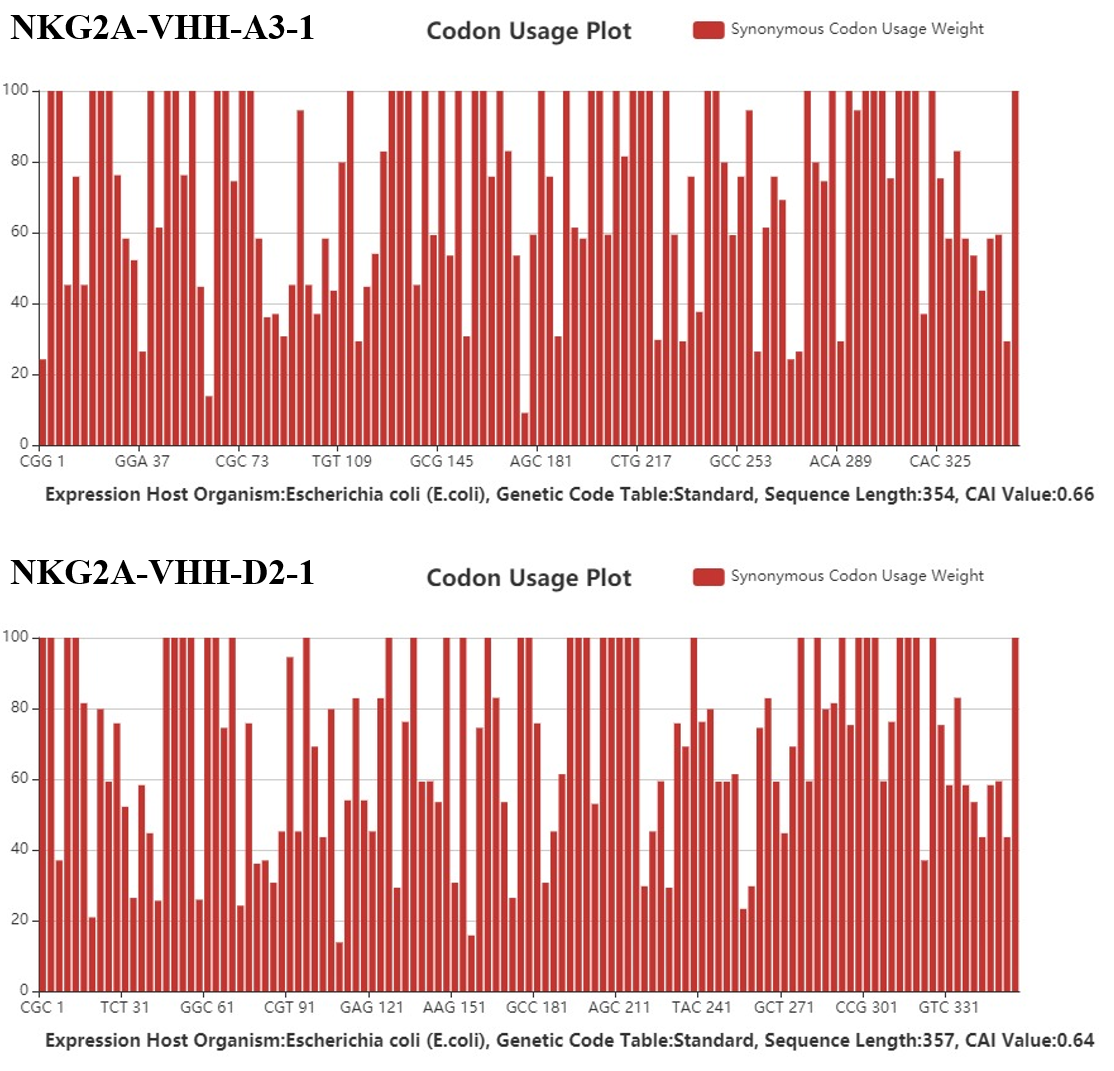


FIG. S3 Rare codon analysis of amino acid sequence expressed by NKG2A-VHH-A3-1 and NKG2A-VHH-D2-1


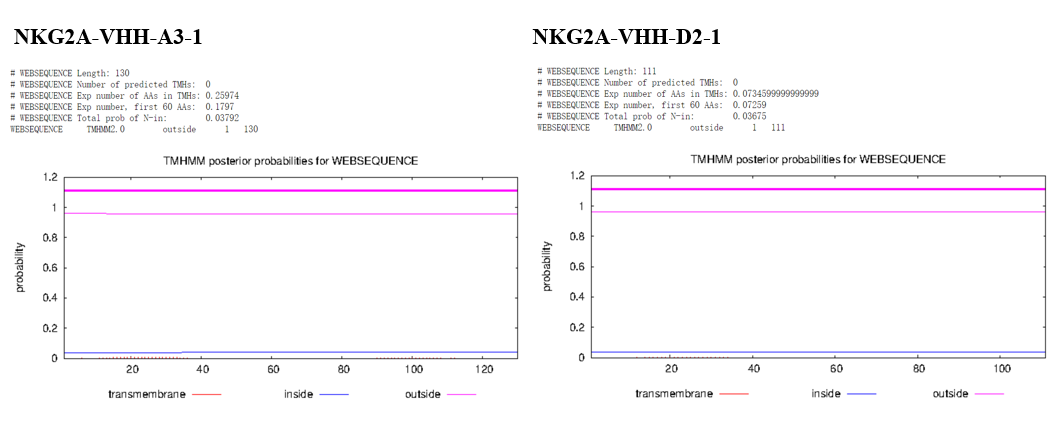


Fig. S4 Analysis of transmembrane region of NKG2A-VHH-A3-1 and NKG2A-VHH-D2-1


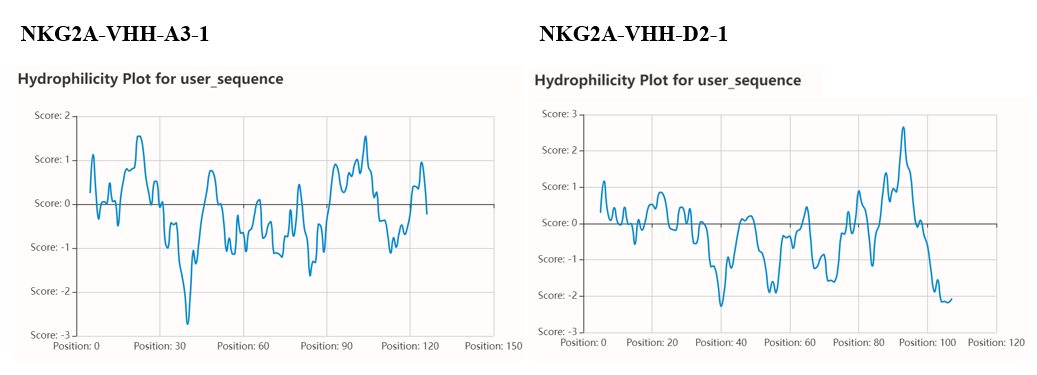


Fig. S5 Hydrophobicity analysis of NKG2A-VHH-A3-1 and NKG2A-VHH-D2-1


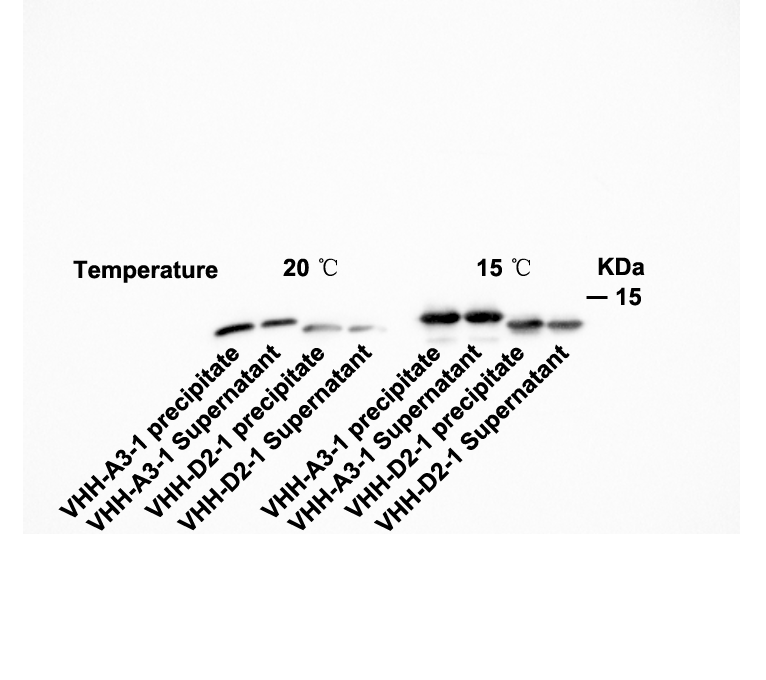


Fig. S6 VHH expression levels of NKG2A-VHH-A3-1 and NKG2A-VHH-D2-1 at 15 ℃ and 20 ℃, respectively


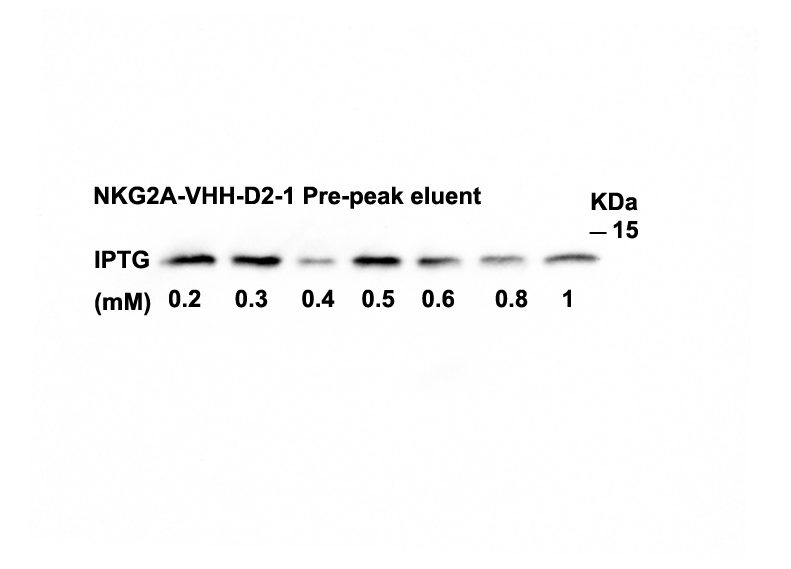


Fig. S7 Expression of VHH in NKG2A-VHH-D2-1 at different concentrations of IPTC（Western blotting results ）


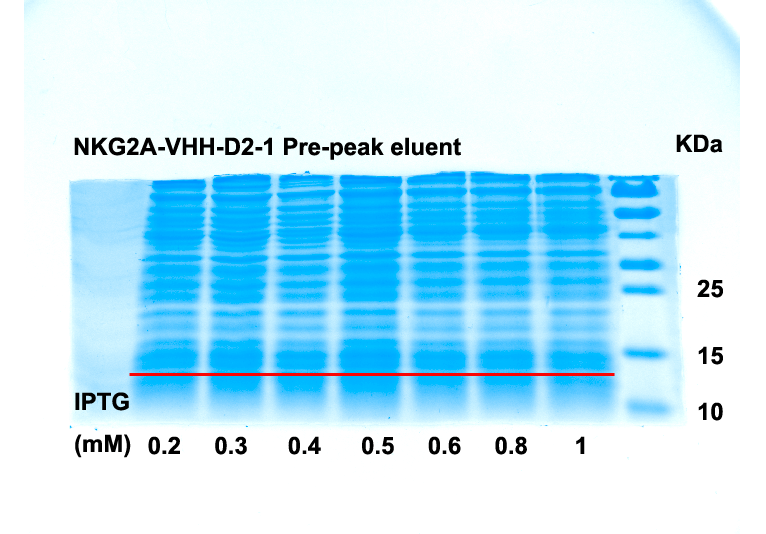


Fig. S8 Expression of VHH in NKG2A-VHH-D2-1 at different concentrations of IPTC（CBB results）

Fig. S9 The effect of NKG2A VHH nanobody with different dose on the growth of melanoma in the Balb/c mouse model.
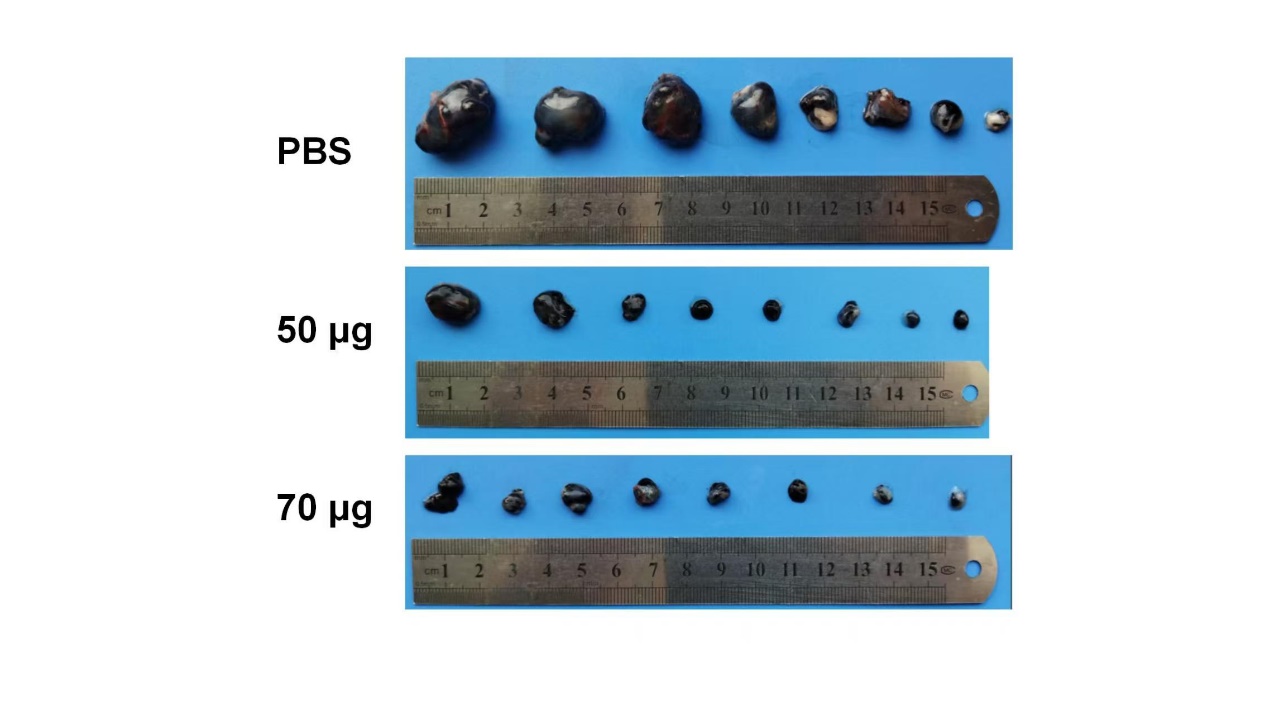


Table S1 The structure of amino acid sequences of NKG2A-VHH-D2-1 single domain antibody

| Region | Sequence |
| --- | --- |
| Frame-1 | QLVESGGGLVQAGRSLRLSCTAS |
| CDR-1 | GFTLEHYA |
| Frame-2 | IGWFRQAPGKGREG |
| CDR-2 | ISCISSNDDTP |
| Frame-3 | YYADFVKGRFTISRDNAKNTVFLQMNSLKCEDIVVVH |
| CDR-3 | SAIIRARGPRSPSPERTT |
